# Case Report: Interim success in treating pyogenic spondylitis caused by Staphylococcus epidermidis using UBE technology: a rare case

**DOI:** 10.3389/fsurg.2026.1847112

**Published:** 2026-06-30

**Authors:** Xiaxing Chen, Jianwei Rao

**Affiliations:** Department of Spine Surgery Jiangshan People’s Hospital, Jiangshan, China

**Keywords:** case report, spondylitis, Staphylococcus epidermidis (S. epidermidis), suppurative infection, UBE

## Abstract

Background Pyogenic spondylitis is a spinal infection caused by non-specific pathogens, with pathological changes typically manifesting as purulent inflammation. The main causative agents include Staphylococcus aureus, Escherichia coli, and others. In recent years, due to factors such as surgical procedures, diabetes mellitus, immunosuppression, and advanced age, the incidence of spinal infections has risen annually—posing significant challenges in diagnosis and treatment. This case report describes the diagnostic and therapeutic experience of a patient with pyogenic spondylitis complicated by diabetes (without obvious immunodeficiency): after poor outcomes with long-term initial therapy, the patient was treated with UBE (Unilateral Biportal Endoscopy) technique. The infection was confirmed to be caused by Staphylococcus epidermidis, with remarkable outcomes observed during follow-up.Case Presentation The patient was a 74-year-old female farmer presenting with pain and weakness in both lower limbs for over 3 months. Her pain was a persistent dull ache—unrelieved by rest, progressively worsening, and scored 7 on the Numerical Rating Scale (NRS). It severely disrupted sleep, requiring opioid analgesics for relief.Based on clinical manifestations, past medical history, laboratory tests, and imaging studies, pyogenic spondylitis was suspected. The patient had undergone a long course of initial anti-infective therapy; while systemic infection was controlled, vertebral infection and intervertebral disc abscess formation compressed nerves—causing intractable lower limb pain. Surgery was thus indicated. The procedure consisted of UBE-assisted posterior lumbar spinal canal exploration and decompression, lumbar lesion resection, and spinal focus debridement. Intraoperatively, multiple tissue samples were collected for bacterial culture and identification. Results confirmed the spinal infection was caused by Staphylococcus epidermidis(S. epidermidis). Subsequently, anti-infective therapy was continued per drug sensitivity testing.At ∼6 months postoperatively, the patient has recovered well: she is afebrile, reports significant pain reduction (NRS score: 2), and shows improvements in laboratory and imaging findings. She remains under continuous follow-up.

## Background

Spinal Infectious Diseases refer to a category of spinal disorders caused by pathogenic microorganisms infecting the vertebral bodies, intervertebral discs, and surrounding soft tissues. In recent years, influenced by factors such as surgical procedures, diabetes, immunosuppression, and advanced age, their incidence rate is approximately 4 per 100,000 individuals (1), showing a year-on-year increase. They have now become a global health concern (2). Based on the type of infecting pathogen and the host's immune response, they are further classified into specific and non-specific spinal infections. Specific spinal infections occur when the body is infected by particular pathogenic microorganisms. The characteristic immune response involves the formation of chronic granulomatous inflammation; hence, they are also termed granulomatous infections. These primarily include: spinal tuberculosis, brucellar spondylitis, fungal spondylitis, and parasitic spondylitis, among others (3–5). Non–specific spinal infections primarily involve pyogenic pathological changes and are therefore also called pyogenic spondylitis. They are mainly caused by bacterial infections such as Staphylococcus aureus and Escherichia coli (6).Spinal infectious diseases often have an insidious onset and a prolonged course. They lack specificity in the early stages, and some patients have no obvious predisposing factors for infection, leading to high rates of missed and misdiagnosis in clinical practice. Although most spinal infectious diseases do not require surgery after conservative treatment, approximately 30% to 50% of patients with Infectious Diseases of the Spine still necessitate surgical intervention (7). Surgical intervention is actively indicated for patients exhibiting progressive worsening of neurological compression symptoms, spinal deformity, or spinal instability; persistent or recurrent sepsis; large abscesses; or failure of conservative treatment after adequate antibiotic therapy (8–10). Surgical approaches include anterior, posterior, and combined anterior-posterior procedures, performed in either a single-stage or staged manner, with or without internal fixation. The anterior approach offers advantages such as clear exposure and thorough debridement of the lesion, adequate decompression, stable bone grafting with reliable support, and preservation of the posterior column structures. It also avoids potential disturbance to the dura mater and spinal canal that may occur during posterior approach debridement (11). The advantage of posterior pedicle screw fixation lies not only in achieving rigid fixation and stabilizing the affected vertebrae but also in correcting deformities. Furthermore, placing internal fixation devices away from the infection site reduces the probability of bacterial adhesion to foreign material, which is more conducive to infection control (12). The combined anterior-posterior approach​ incorporates the benefits of both anterior and posterior surgeries (13). However, it inevitably results in greater surgical trauma and longer operative times. In recent years, reports on using minimally invasive techniques to treat IDS have gradually increased, primarily involving debridement, drainage, and catheter lavage, which are suitable for cases with smaller lesions. Unilateral Biportal Endoscopy(UBE)​technology has emerged and is increasingly used to treat lumbar degenerative diseases (LDD) such as Lumbar Disc Herniation (LDH), Lumbar Spinal Stenosis (LSS), and Degenerative Lumbar Spondylolisthesis (DLS) (14, 15). This technique establishes two relatively independent channels—an observation channel and a working channel—through two small incisions. The observation channel accommodates an endoscopic irrigation system for monitoring and continuously irrigating the surgical area, while the working channel allows for the insertion of various surgical instruments. With its clinical application and refinements, its indications have continuously expanded, achieving success in areas such as debridement, alleviating back pain, and obtaining bacteriological diagnoses for lumbar infectious spondylodiscitis (16). This article presents a case of pyogenic spondylitis caused by Staphylococcus epidermidistreated with UBE technology, hoping to provide a reference for the diagnosis and treatment of spinal infections in clinical practice.

## Presentation of case

**Figure 1:**
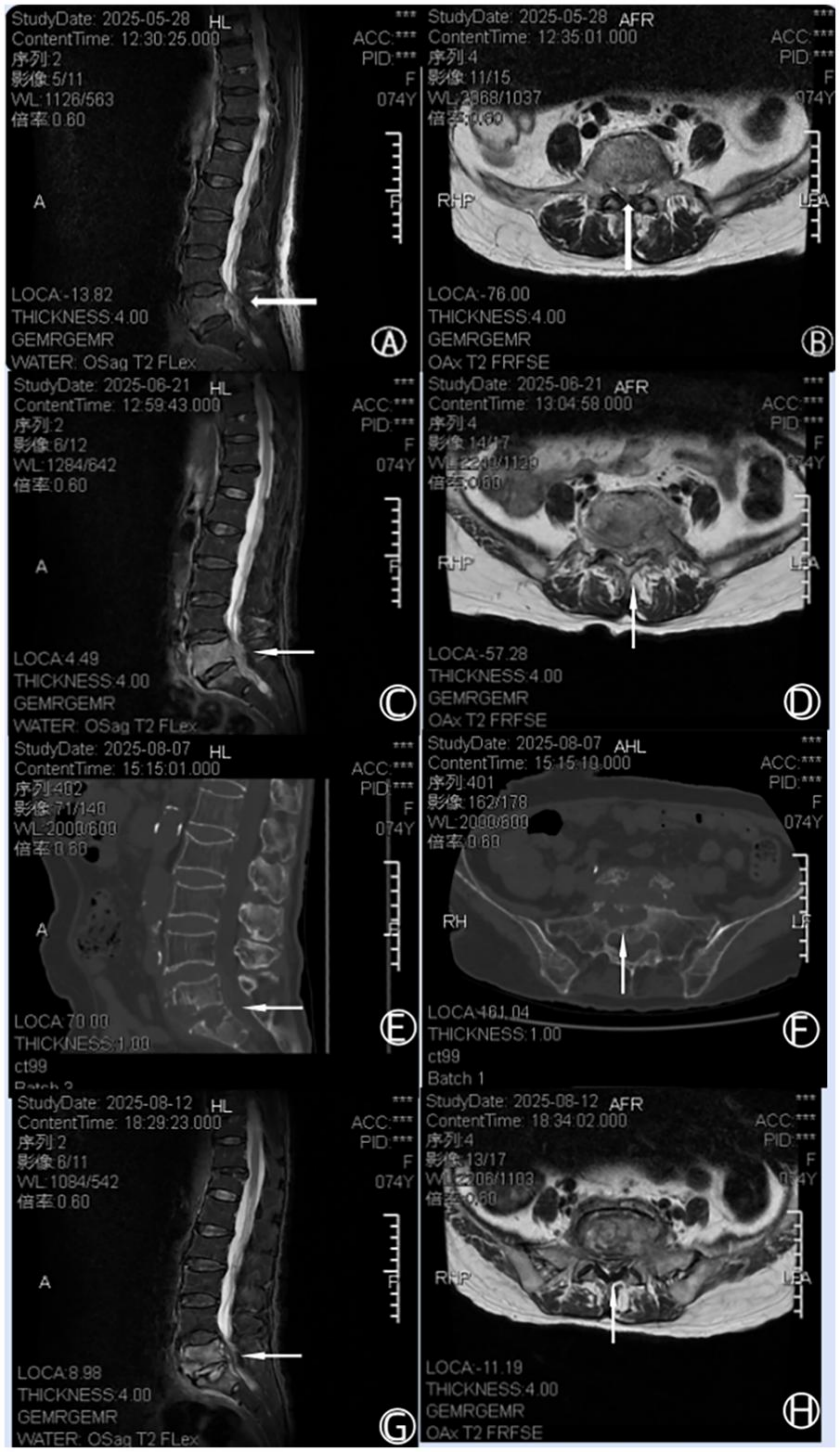
Preoperative lumbar MRI and CT showing progression of the infection at the L5 vertebra with epidural abscess formation.

A 74-year-old female patient was initially admitted to the Endocrinology Department of our hospital on May 16, 2025, due to “elevated blood glucose for over 10 years, accompanied by chills and fever for half a day.” Blood cultures obtained on May 19, 2025, identified a Staphylococcus aureus infection. During hospitalization, she received anti-infective therapy with piperacillin-tazobactam (May 16–23), vancomycin (May 23–31), and levofloxacin injection (May 31–June 1). Due to concurrent complaints of low back and leg pain, an MRI was performed, revealing infection at the L5 vertebra with epidural abscess formation (Figures 1A,B). Subsequently, the patient requested transfer to a higher-level hospital. There, a diagnosis of infective endocarditis was also considered. Starting June 5, she received treatment with cefazolin 1 g every 8 h + rifampicin 300 mg twice daily. Following this, her inflammatory markers showed improvement. She was later readmitted to our hospital. From June 10 to June 26, she continued the same regimen of cefazolin + rifampicin capsules. However, a follow-up C-reactive protein (CRP) test showed a slight increase, and her lower limb pain showed no significant improvement. A repeat MRI suggested possible progression of the L5 vertebral infection and epidural abscess (Figures 1C,D). Considering the suboptimal response to cefazolin, the anti-infective regimen was changed from June 26 to July 4 to linezolid tablets + rifampicin capsules. On July 4, the patient developed nausea, vomiting, and severe hyponatremia with hypochloremia, suspected to be adverse effects of linezolid. The linezolid was discontinued. From July 4 to July 7, anti-infective therapy was switched to levofloxacin tablets + rifampicin capsules. During this period, the patient became afebrile, with resolution of nausea and vomiting, and improvement in electrolyte imbalances. However, a subsequent CRP test showed a slight rise again. The regimen was then changed to amoxicillin-clavulanate tablets + rifampicin capsules. Concurrently, she received supportive treatments including analgesia, glycemic control, calcium supplementation, diuretics, cardiac function support, and fluid/nutritional therapy. Her condition improved, and she was discharged with instructions to continue the same oral antibiotics. But,she was readmitted to the Infectious Diseases Department of our hospital on August 4, 2025, due to fatigue and poor appetite, with an admitting diagnosis of “Staphylococcus aureus septicemia”. Following consultation with our department, a lumbar CT scan was performed, revealing bone destruction at the L5 and S1 vertebrae (Figures 1E,F). Anti-infective therapy with the prior regimen (amoxicillin-clavulanate + rifampicin) was continued. By August 8, follow-up tests showed that her white blood cell count, CRP, and procalcitonin (PCT) had normalized. As the intended treatment course for infective endocarditis was deemed complete, rifampicin capsules were discontinued, and she continued on amoxicillin-clavulanate alone. Due to persistent bilateral lower limb pain and weakness, she was transferred to our department on August 14 for further management of “vertebral infection.” Physical examination revealed the patient in a forced posture. There was no obvious tenderness or percussion pain over the lumbar spine, but deep pressure elicited discomfort in the lower lumbar region. Lumbar flexion, extension, and rotation were limited. Muscle strength testing of the left lower limb showed: hip flexion, knee extension, ankle dorsiflexion, and great toe dorsiflexion at grade 4; ankle plantar flexion at grade 4-. There was decreased superficial sensation over the posterolateral aspect of the left lower limb. Distal circulation appeared normal. The straight leg raise test was positive on the left side. As surgical intervention was decided upon, a preoperative lumbar MRI was repeated (Figures 1G,H) for updated assessment.

**Figure 2:**
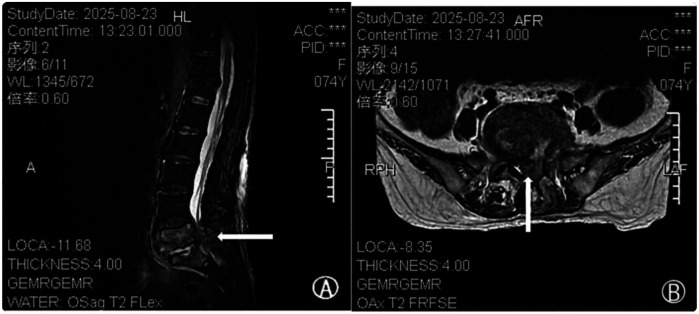
Lumbar MRI showing reduction of the intraspinal abscess.

On August 17, 2025, from 09:45 to 11:50, the patient underwent a “Unilateral Biportal Endoscopic (UBE) Posterior Lumbar Spinal Canal Exploration and Decompression, Lumbar Lesion Excision, and Endoscopic Spinal ” under general anesthesia via a left-sided approach. Intraoperative Findings: Significant hypertrophy of the facet joints was observed. After partial resection of the inferior margin of the left L5 lamina, the superior margin of the left S1 lamina, and part of the facet joints, the ligamentum flavum was exposed at its origin and insertion points, then resected to reveal the dural sac. Upon dissection along the dural sac to mobilize the left nerve root, significant intraspinal granulation tissue proliferation, adhesion of surrounding soft tissues, and congestion and edema of the nerve root were noted. Destructive changes were evident in the inferior endplate of the L5 vertebra and the superior endplate of the S1 vertebra, surrounded by substantial necrotic tissue and sequestra. A bulging annulus fibrosus and herniated nucleus pulposus were compressing the S1 nerve root. Surgical Procedure: The herniated nucleus pulposus was removed. Adherent tissues were carefully dissected. Necrotic and hyperplastic soft tissues were thoroughly curetted. Following resection of the necrotic intervertebral disc, the disc space was entered to debride sequestra from the superior and inferior vertebral bodies. Specimens of the infected and necrotic tissues were collected and sent for bacterial culture and antimicrobial susceptibility testing. Completion: After copious irrigation of the infected disc space, local radiofrequency was applied for hemostasis. Upon confirming restoration of normal pulsation in the dural sac, absence of significant nerve root compression, and no residual necrotic tissue, a drainage tube was placed. The incision was then closed to conclude the procedure. Postoperative Course: The patient reported significant improvement in lower limb pain postoperatively. Anti-infective therapy was continued. A follow-up lumbar MRI performed after her condition stabilized showed reduction of the intraspinal abscess, with a possibility of partial hematoma formation (Figure 2). On August 20, culture and identification of the surgical specimen confirmed an infection with Staphylococcus epidermidis, which was sensitive to linezolid. Discharge and Follow-up: The patient was discharged with a prescription for oral linezolid tablets to continue anti-infective treatment and instructed to return for regular outpatient follow-up examinations.

## Discussion

Pyogenic spondylitis exhibits geographical variations in the reported prevalence of causative bacteria. Nevertheless, Gram-positive cocci, predominantly Staphylococcus aureus, consistently represent the most frequently isolated pathogens globally. The primary routes of infection include hematogenous spread, contiguous extension from adjacent foci, and direct inoculation during spinal procedures (17). Common primary sources of infection encompass the genitourinary tract, skin and soft tissues, and intravascular devices (18). Staphylococcus epidermidis, a commensal bacterium colonizing human skin and mucosal surfaces, is generally considered non-pathogenic. However, it functions as an opportunistic pathogen. Breaches in the skin or mucosal barrier due to medical interventions, immunosuppression following major surgery, or underlying immunocompromising conditions can facilitate its entry into the bloodstream and subsequent infection. At the same time, specimen contamination or a polymicrobial disease may also lead to the growth of Staphylococcus epidermidis in the specimen. In this case, the patient has suffered from diabetes for many years. Being elderly and frail, with multiple comorbidities, and after undergoing prolonged antibiotic treatment for infections, there is a possibility of bacterial imbalance in the body. Staphylococcus epidermidis, which normally resides on the skin and mucous membranes, can invade the body when resistance decreases, leading to what is known as an opportunistic infection. In addition, pyogenic spondylitis has an long incubation period and the infection site is deep, resulting in atypical clinical manifestations at the beginning. Laboratory inflammatory markers lack specificity, and early imaging studies frequently yield low sensitivity. While later imaging may reveal signs of infection, identifying the precise pathogen remains challenging. Histoopathological examination requires advanced puncture techniques and equipment. It carries certain risks associated with puncture, and there is a possibility of specimen contamination. The accuracy of the test is also affected significantly by the quality of the specimen. Although microbiological culture constitutes the diagnostic gold standard, both blood and tissue cultures demonstrate low positivity rates (19), making clinical diagnosis difficult and leading to a high rate of misdiagnosis or missed diagnoses. Blood cultures taken on May 19 during the early stages of the disease detected Staphylococcus aureus. The drug sensitivity test showed that the bacterium was sensitive to vancomycin. Therefore, vancomycin was immediately administered for anti-infection treatment. After treatment, the inflammatory markers improved. Later, due to the patient's symptoms of lower back and leg pain, a lumbar MRI was performed, which revealed a concurrent lumbar infection. However, at this early stage of the infection and with antibiotics already in use, the positive rate of percutaneous biopsy would be low. Forcing a biopsy would likely not lead to the identification of the pathogen and could instead increase the patient's suffering and risk. Thus, performing a CT-guided biopsy at an early stage is considered a procedure with “high risk and low benefit”.

**Figure 3:**
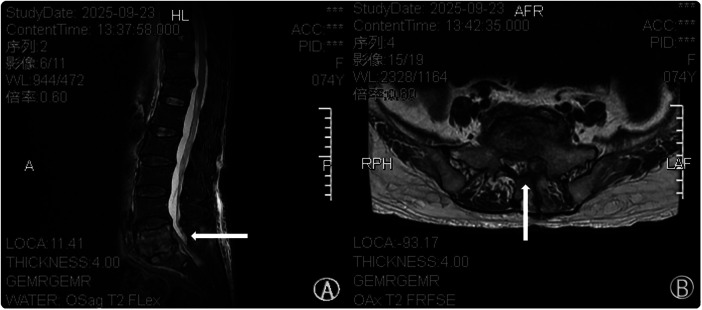
MRI showed a significant reduction in spinal abscess after more than a month of surgery.

**Figure 4:**
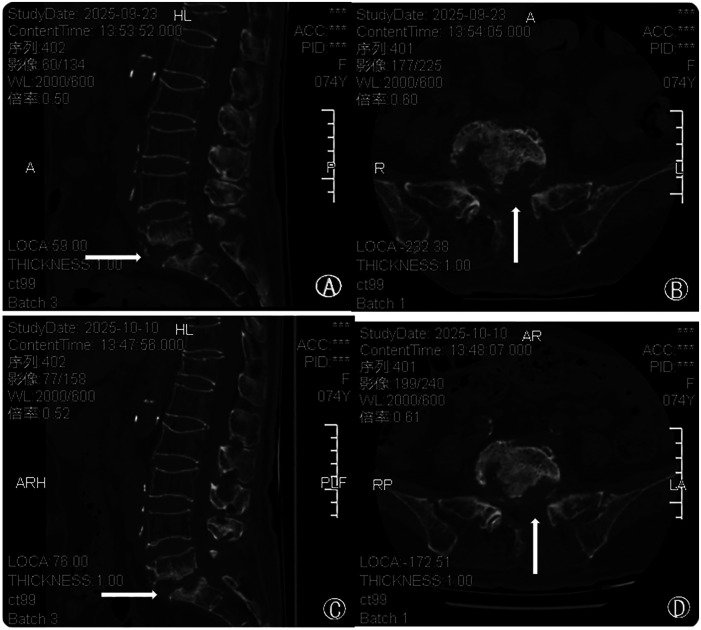
CT scan showed improvement in L5 and S1 vertebral bone defects compared to before.

After prolonged treatment with vancomycin and other antibiotics, the patient's inflammatory markers decreased, the blood cultures drawn on June 6, June 17, and August 11 all returned negative results. It can be preliminarily concluded that the treatment was effective against bacteremia caused by Staphylococcus aureus. Therefore, an appropriate timing for CT-guided puncture biopsy could not be determined in the early stages. However, as the patient's inflammatory indicators remained elevated and her back and leg pain did not improve, CT-guided puncture biopsy became ineffective later on. Treatment became absolutely necessary; the patient needed not only a diagnosis but also relief from nerve compression and removal of the infected tissue. UBE allows for “diagnosis + treatment” in one procedure: under direct visualization through the endoscope, doctors can obtain sufficient tissue from the most affected area. This is much more accurate than using a thin needle under CT guidance, enabling comprehensive culture tests, pathological analysis, and next-generation sequencing (NGS). Unfortunately, due to financial reasons, the patient did not undergo NGS testing. With UBE, once the sample is collected, the abscessed tissue and inflamed granulation can be removed using a curette and burr, thereby relieving nerve compression. The area is then rinsed thoroughly with saline. Consequently, we employed UBE to perform thorough debridement, irrigation, and spinal canal decompression, while simultaneously obtaining deep tissue specimens for diagnostic purposes. Ensuring that tissue samples are collected in the operating room significantly reduces the risk of contamination. Additionally, This minimally invasive approach allowed for effective surgical intervention within a controlled trauma framework, potentially shortening the recovery period for this immunocompromised patient and ensuring favorable short-term outcomes. At this point, the emergence of UBE technology is not a “compromise on diagnostic methods”, but a strategic upgrade from “simple antibacterial” to “minimally invasive debridement + precise diagnosis” in response to changes in the condition. Serial postoperative imaging follow-up (Figures 3, 4) indicates a trend toward resolution of the local infective focus. However, the most recent imaging still reveals a localized bony defect, suggesting relative segmental instability. Considering that the patient had a prolonged period of local infection, implanting internal fixation devices in the first phase would not only increase the degree of local damage and prolong surgery time but also make it difficult to control the infection, as the implanted devices would act as foreign objects near the infected area, potentially leading to further spread of the infection. Therefore, internal fixation was not performed in the first phase. Additionally, during the surgery, we made every effort to preserve the facet joints, thereby minimizing any disruption to lumbar stability. After the surgery, with the support of a lumbar brace, the overall stability of the spine remained satisfactory. The patient is currently continuing conservative treatment. During subsequent follow-up, close monitoring of systemic inflammatory markers and serial local imaging remains imperative. If the patient experiences further progression of local bone resorption, along with obvious symptoms and radiological signs of lumbar instability, a second-stage bone grafting procedure with internal fixation can be considered. However, at present, the patient's local condition is improving, and there are no signs of significant spinal instability. With the protection provided by the lumbar brace, internal fixation surgery is not deemed necessary for now. It can be seen that the combination of UBE debridement and the subsequent definitive microbiological diagnosis has proven crucial in guiding appropriate antimicrobial therapy. The patient has recovered satisfactorily, marking a successful interim outcome in her management, and she is experiencing a promising short-term outcome. With concurrent control of her underlying conditions, this treatment strategy has substantially improved her quality of life.

Reviewing the patient's previous treatment history, Staphylococcus aureus was initially identified, and active treatment was administered. Subsequent blood cultures yielded negative results in all three tests. However, the postoperative specimen culture confirmed Staphylococcus epidermidis. It is almost certain that it was a primary infection caused by Staphylococcus aureus, followed by a secondary infection (opportunistic infection) triggered by Staphylococcus epidermidis. Of course, the possibility of a co-infection with both of the first two types of bacteria cannot be ruled out. It's possible that this case also represents a polymicrobial disease.

## Conclusion

In summary, spinal infectious diseases are typically characterized by an insidious onset, prolonged latency, high rates of misdiagnosis or delayed diagnosis, and significant treatment challenges. They are frequently observed as postoperative infections. The present case is relatively rare, involving an infection initially suspected to be secondary to Staphylococcus aureusbacteremia but later confirmed to be caused by Staphylococcus epidermidis. Through the application of UBE technology, along with recent follow-up and a comprehensive review of the patient's entire treatment course, this case provides an in-depth analysis and summary, offering a novel perspective for clinical practice. In clinical settings, for elderly patients presenting with low back pain, leg pain, or limb weakness—particularly those with multiple comorbidities—if conventional treatments yield suboptimal results or symptoms recur and progress, timely repeat MRI is warranted to assess the extent of lesion invasion, nerve root compression, or spinal canal occupancy. If infection is suspected, further histopathological examination and pathogen culture should be pursued to enhance diagnostic accuracy. Antibiotic therapy should be guided by pathogen identification and antimicrobial susceptibility testing. In the absence of microbiological confirmation, empirical broad-spectrum antibiotics covering likely pathogens such as S. epidermidis, Streptococcusspecies, and Escherichia coli should be initiated. For patients who show poor response to anti-infective therapy or meet surgical indications, timely surgical intervention is crucial. The advantages of the UBE surgical technique provide spine surgeons with an additional valuable option in their therapeutic arsenal.

## Data Availability

The original contributions presented in the study are included in the article/Supplementary Material, further inquiries can be directed to the corresponding author.
